# Supplementary material for: Enhanced biomass and thermotolerance of Arabidopsis by SiERECTA isolated from Setaria italica L
Source: PeerJ. 2022 Dec 1;10:e14452. doi: 10.7717/peerj.14452 (PMC9744159; doi:10.7717/peerj.14452)
Supplement: Supplemental Information 1 [file peerj-10-14452-s001.docx]

**Annex 1 Table S1** The URLs list of biological database

| No. | Website | URLs of database |
| --- | --- | --- |
| 1 | Phytozome v12.1 | <https://phytozome.jgi.doe.gov/pz/portal.html> |
| 2 | NCBI | <https://www.ncbi.nlm.nih.gov> |
| 3 | PROSITE | <https://prosite.expasy.org> |
| 4 | SMART | http://smart.embl-heidelberg.de |
| 5 | ProtParam | <https://web.expasy.org/protparam/> |
| 6 | GSDS2.0 | <http://gsds.gao-lab.org/> |
| 7 | PlantCARE | <http://bioinformatics.psb.ugent.be/webtools/plantcare/html/> |
| 8 | Plant-mPLoc | <http://www.csbio.sjtu.edu.cn/cgi-bin/PlantmPLoc.cgi> |
| 9 | MEME Suite 5.4.1 | <https://meme-suite.org/meme/tools/meme> |
