# Supplementary material for: Enhanced biomass and thermotolerance of Arabidopsis by SiERECTA isolated from Setaria italica L
Source: PeerJ. 2022 Dec 1;10:e14452. doi: 10.7717/peerj.14452 (PMC9744159; doi:10.7717/peerj.14452)
Supplement: Supplemental Information 2 [file peerj-10-14452-s002.docx]

**Annex 2 Table S2** NCBI accession numbers of proteins in phylogenetic tree

| No. | ER family proteins | Plant Species | NCBI accession number | Classification |
| --- | --- | --- | --- | --- |
| Group I | | | | |
| 1 | *SiER1_X1* | *Setaria italica* L. | XP_014660111.1 | monocots |
| 2 | *SiER1_X2* | *Setaria italica* L. | XP_014660109.1 | monocots |
| 3 | *SiER1_X3* | *Setaria italica* L. | XP_014660108.1 | monocots |
| 4 | *SiER1_X4* | *Setaria italica* L. | Annex 4 (OP492075) | monocots |
| 5 | *SiER1_X5* | *Setaria italica* L. | XP_034576484.1 | monocots |
| 6 | *SiER1_X6* | *Setaria italica* L. | XP_034576491.1 | monocots |
| 7 | OsERECTA | *Oryza sativa Japonica Group* L. | XP_015623966.1 | monocots |
| Group II | | | | |
| 8 | *SiER4_X1* | *Setaria italica* L. | XP_004964941.1 | monocots |
| 9 | *SiER4_X2* | *Setaria italica* L. | XP_004964942.1 | monocots |
| 10 | *SbER10_X1* | *Sorghum bicolor* L. | XP_002438023.1 | monocots |
| 11 | *ZmERECTA* | *Zea mays* L. | NP_001345763.1 | monocots |
| 12 | *AetERECTA* | *Aegilops tauschii subsp. t.auschii* L. | XP_020193416.1 | monocots |
| 13 | *TaERECTA* | *Triticum aestivum* L | AFJ38187.2 | monocots |
| 14 | *HvERECTA* | *Hordeum vulgare subsp. vulgare* L. | AKU38976.1 | monocots |
| 15 | *BdERECTA* | *Brachypodium distachyon* L. | XP_003564133.1 | monocots |
| Group III | | | | |
|  | *GmERECTA* | *Glycine max* L. | XP_003544548.1 | dicots |
|  | *VvERECTA* | *Vitis vinifera* L. | XP_002280069.2 | dicots |
| Group IV | | | | |
|  | *SiERL4* | *Setaria italica* L. | XP_004964421.1 | monocots |
|  | *AtERECTA* | *Arabidopsis thaliana* L. | NP_180201.1 | dicots |
|  | *AtERL_1* | *Arabidopsis thaliana* L. | NP_001190595.1 | dicots |
|  | *AtERL_2* | *Arabidopsis thaliana* L. | NP_001331523.1 | dicots |

Note: OP492075 is a GenBank accession number for *SiER1_X4*
