# Supplementary material for: Enhanced biomass and thermotolerance of Arabidopsis by SiERECTA isolated from Setaria italica L
Source: PeerJ. 2022 Dec 1;10:e14452. doi: 10.7717/peerj.14452 (PMC9744159; doi:10.7717/peerj.14452)
Supplement: Supplemental Information 3 [file peerj-10-14452-s003.docx]

**Annex 3 Table S3** The sequence of primers related to PCR amplification

| Name | Pair of forward and reverse primers (5’-3’) | Tm | Size | Restriction Enzyme | Note |
| --- | --- | --- | --- | --- | --- |
| *SiER1_X4-F2* | CCTCCTCGCGAATGCCACTGCTGCC | 65℃ | 3058 bp | *－* | *SiER1_X4* gene isolation |
| *SiER1_X4-R2* | TCGCAGGCTGAACTCTTGAGACGATCG |  |  |  |  |
| *SiER4_X1-F3* | GGCAGGCTTGCTGCAGCACGCTTCG | 65℃ | 3113 bp |  | *SiER4_X1* gene isolation |
| *SiER4_X1-R3* | TTCACTATCTGCACCCTCCACCGCCGC |  |  |  |  |
|  |  |  |  |  |  |
| *SiER1_X4-gfpF1* | TATCTCTAGA **GGATC**C ATGACCCGCCTCCTCCGGGC | 62℃ | 3002 bp | *BamH* I | Subcellular localization |
| *SiER1-X4- gfpR1* | TGCTCACCAT **GGATCC** TTCTGTGTTCCGTGATATCACCTCG |  |  |  |  |
| *SiER4_X1-gfpF1* | TATCTCTAGA **GGATCC** ATGCTTGTCCGCAGCTCAGTG | 61℃ | 3020 bp |  |  |
| *SiER4-X1- gfpR1* | TGCTCACCAT **GGATCC** CTCCGTGTTCTGCGAGATGG |  |  |  |  |
|  |  |  |  |  |  |
| *SiER1_X4-1302F1* | GGGACTCTTGA **CCATGG** ATGACCCGCCTCCTCCGGGC | 64℃ | 3003 bp | *Nco* I | Transformation of Arabidopsis |
| *SiER1-X4-1302R1* | TCAGATCTAC **CCATGG** TTCTGTGTTCCGTGATATCACCTCG |  |  |  |  |
| *SiER4_X1-1302F1* | GGGACTCTTGA **CCATGG** ATGCTTGTCCGCAGCTCAGTG | 62℃ | 3021 bp |  |  |
| *SiER4_X1-1302R1* | TCAGATCTAC **CCATGG** CTCCGTGTTCTGCGAGATGG |  |  |  |  |
|  |  |  |  |  |  |
| *SiER1_X4 –qRTF2* | CTATTGGCGTGGTTCTCGTTCT | 58℃ | 232bp | *－* | qRT-PCR for *SiERs* expression |
| *SiER1_X4 –qRTR2* | CTTGGGAGGAACACTGCTTGAT |  |  |  |  |
| *SiER4_X1-qRTF1* | GGCTGCGATACTTGGCATTG |  | 126 bp | *－* |  |
| *SiER4_X1-qRTR1* | CCATTGCTCACTGGTTTGCTT |  |  |  |  |
| *SiActin-qRTF1* | TGGTATGGAGTCGCCTGGAATC |  | 114 bp | *－* |  |
| *SiActin-qRTR1* | GCCACCGCTGAGCACAATGTTA |  |  |  |  |
|  |  |  |  |  |  |
| *AtBI1-qRTF1* | CAGGGAGTATCTCTACCTTGGA | 55℃ | 152bp | *－* | qRT-PCR for stress-related genes expression |
| *AtBI1-qRTR1* | CCACCATGTATCCCACAAAGA |  |  |  |  |
| *AtHSFA1a-qRTF2* | TGAGGTTGGGAAATTTGGGTTA |  | 106bp | *－* |  |
| *AtHSFA1a-qRTR2* | TGTTGTTTGTTGCTGCTGGCGT |  |  |  |  |
| *AtActin-qRTF5* | ACCACTACCGCAGAACGGGAAA |  | 198bp | *－* |  |
| *AtActin-qRTR5* | GAGCGATGGCTGGAACAGAACC |  |  |  |  |
