# Supplementary material for: Enhanced biomass and thermotolerance of Arabidopsis by SiERECTA isolated from Setaria italica L
Source: PeerJ. 2022 Dec 1;10:e14452. doi: 10.7717/peerj.14452 (PMC9744159; doi:10.7717/peerj.14452)
Supplement: Supplemental Information 4 [file peerj-10-14452-s004.pdf]

Annex 4 Figure S1 The coding sequence of SiER1_X4 gene GenBank accession number: OP492075

SiER1_X4 (1-2973)

```
Total amino acid number:990,MW=107753
Max ORF starts at AA pos 1(may be DNA pos 1)for 990 AA(2970 bases),MW=107753

                 10        20        30        40        50        60        70        80        90
1        ATGACCCGCCTCCTCCGGGCCCTCGCCGCCTTCCTCCTCCTCGCGGCCGTCGCCGTCGCCGACGACGGGGCAACACTGCTGGAGATCAAG
1         M  T  R  L  L  R  A  L  A  A  F  L  L  L  A  A  V  A  V  A  D  D  G  A  T  L  L  E  I  K

                100       110       120       130       140       150       160       170       180
91       AAATCCTTCAGCAACGGAGGCAACGCGCTGCACGATTGGTCCGGCGAGGGCGCATCGCCGACCTACTGCTCGTGGCGCGGCGTGCTATGC
31        K  S  F  S  N  G  G  N  A  L  H  D  W  S  G  E  G  A  S  P  T  Y  C  S  W  R  G  V  L  C

                190       200       210       220       230       240       250       260       270
181      GACAACGTCACCTTCGCTGTTGCGGCGCTCAACCTTTCAGGGCTCAATCTCGAGGGTGAAATCTCACCGGCCATCGGGAGCCTGAAACGT
61        D  N  V  T  F  A  V  A  A  L  N  L  S  G  L  N  L  E  G  E  I  S  P  A  I  G  S  L  K  R

                280       290       300       310       320       330       340       350       360
271      GTTATCTCAATAGATTTGAAGTCTAATGGACTTTCCGGGCAGATCCCTGATGAGATTGGCGATTGTTCGTTGCTTGAAACTTTGGACTTG
91        V  I  S  I  D  L  K  S  N  G  L  S  G  Q  I  P  D  E  I  G  D  C  S  L  L  E  T  L  D  L

                370       380       390       400       410       420       430       440       450
361      TCCTCTAACAATCTAGAAGGAGACATACCATTCTCCATATCTAAGCTCAAGCACCTTGAGAACCTGATTTTGAAGAACAACCAGCTGGTT
121       S  S  N  N  L  E  G  D  I  P  F  S  I  S  K  L  K  H  L  E  N  L  I  L  K  N  N  Q  L  V

                460       470       480       490       500       510       520       530       540
451      GGGGTGATACCATCAACACTATCTCAACTTCCTAATTTGAAGATATTGGACTTGGCTCAAAACAAGCTAAGTGGTGAAATTCCAAACCTA
151       G  V  I  P  S  T  L  S  Q  L  P  N  L  K  I  L  D  L  A  Q  N  K  L  S  G  E  I  P  N  L

                550       560       570       580       590       600       610       620       630
541      ATATATTGGAATGAGGTTCTTCAATACTTGGGATTGCGGAGCAATAATTTAGAAGGAAGCCTCTCTCCGGATATGTGCCAGTTAACTGGC
181       I  Y  W  N  E  V  L  Q  Y  L  G  L  R  S  N  N  L  E  G  S  L  S  P  D  M  C  Q  L  T  G

                640       650       660       670       680       690       700       710       720
631      CTGTGGTACTTTGATGTGAAGAATAATAGCTTGATGGGTACGATACCAGAAACCATAGGGAACTGTACCAGCTTTCAGGTCTTGGATTTG
211       L  W  Y  F  D  V  K  N  N  S  L  M  G  T  I  P  E  T  I  G  N  C  T  S  F  Q  V  L  D  L

                730       740       750       760       770       780       790       800       810
721      TCAAACAATCAGCTTACTGGAGAAATCCCATTCAATATTGGTTTCCTGCAAGTGGCTACGCTGTCTTTGCAAGGGAACAAGTTCTCTGGC
241       S  N  N  Q  L  T  G  E  I  P  F  N  I  G  F  L  Q  V  A  T  L  S  L  Q  G  N  K  F  S  G

                820       830       840       850       860       870       880       890       900
811      CCAATTCCATCAGTGATTGGCCTTATGCAGGCGCTTGCAGTGCTCGATCTGAGTTTCAATGAGCTATCTGGACCAATACCATCTATACTG
271       P  I  P  S  V  I  G  L  M  Q  A  L  A  V  L  D  L  S  F  N  E  L  S  G  P  I  P  S  I  L

                910       920       930       940       950       960       970       980       990
901      GGCAACTTGACATACACTGAGAAATTATACCTGCAAGGCAATAGGCTAACTGGATCGATACCTCCAGAGCTTGGTAATATGACGACACTG
301       G  N  L  T  Y  T  E  K  L  Y  L  Q  G  N  R  L  T  G  S  I  P  P  E  L  G  N  M  T  T  L

               1000      1010      1020      1030      1040      1050      1060      1070      1080
991      CATTACCTTGAACTGAATGACAATCTATTGACTGGGTTCATTCCTCCTGATCTTGGGAAGCTCACAGAATTGTTTGACTTGAACCTTGCA
331       H  Y  L  E  L  N  D  N  L  L  T  G  F  I  P  P  D  L  G  K  L  T  E  L  F  D  L  N  L  A

               1090      1100      1110      1120      1130      1140      1150      1160      1170
1081     AACAACAACCTTGGAGGACCTATCCCTGATAATATAAGTTCATGCATAAATCTCATTAGTTTGAATGCTTATGACAATAAATTAAATGGA
361       N  N  N  L  G  G  P  I  P  D  N  I  S  S  C  I  N  L  I  S  L  N  A  Y  D  N  K  L  N  G

               1180      1190      1200      1210      1220      1230      1240      1250      1260
1171     ACCATTCCACGTTCATTTCAAAAGCTCGAGAGTCTGACTTATCTGAATCTGTCATCAAATCATCTCAGTGGAGCACTTCCGATTGAGGTA
391       T  I  P  R  S  F  Q  K  L  E  S  L  T  Y  L  N  L  S  S  N  H  L  S  G  A  L  P  I  E  V

               1270      1280      1290      1300      1310      1320      1330      1340      1350
1261     GCAAGAATGAGAAATTTAGACACACTGGACTTATCCTGTAACATGATCACTGGTTCAATTCCCTCAGCTATTGGAAGACTAGAGCATCTT
421       A  R  M  R  N  L  D  T  L  D  L  S  C  N  M  I  T  G  S  I  P  S  A  I  G  R  L  E  H  L

               1360      1370      1380      1390      1400      1410      1420      1430      1440
1351     CTGAGGCTCAATTTAAGCAAAAATGCTCTGGTTGGACACATTCCTGCTGAGTTTGGGAACTTGAGGAGCATCATGGAGATCGATTTGTCC
451       L  R  L  N  L  S  K  N  A  L  V  G  H  I  P  A  E  F  G  N  L  R  S  I  M  E  I  D  L  S

               1450      1460      1470      1480      1490      1500      1510      1520      1530
1441     AGTAACTACCTCCGTGGCCTGATTCCACAAGAGGTTGGGATGCTACAAAATCTGATACTGTTAAAATTAGAAAATAATAGTATTACTGGA
481       S  N  Y  L  R  G  L  I  P  Q  E  V  G  M  L  Q  N  L  I  L  L  K  L  E  N  N  S  I  T  G

               1540      1550      1560      1570      1580      1590      1600      1610      1620
1531     GATGTCTCACCACTCACTAACTGTTTCAGTCTCAATAACTTAAATGTATCATACAACGACCTTGCTGGTATTGTACCTACAGACAACAAC
511       D  V  S  P  L  T  N  C  F  S  L  N  N  L  N  V  S  Y  N  D  L  A  G  I  V  P  T  D  N  N

               1630      1640      1650      1660      1670      1680      1690      1700      1710
1621     TTCTCACGATTTTCACCTGACAGCTTCTTGGGTAACCCTGGACTTTGTGGCTATTGGCGTGGTTCTCGTTCTTCGTGCTCTCCTTTATCA
541       F  S  R  F  S  P  D  S  F  L  G  N  P  G  L  C  G  Y  W  R  G  S  R  S  S  C  S  P  L  S

               1720      1730      1740      1750      1760      1770      1780      1790      1800
1711     TCCAGTATTGAGCGCAAAAGGAGATCATCTATATCAAAGGCTGCATTTCTTGGTATTGGTGTTGGTGGCTTGTTATCCTGCTCGTTATC
571       S  S  I  E  R  K  R  R  S  S  I  S  K  A  A  F  L  G  I  G  V  G  G  L  V  I  L  L  V  I

               1810      1820      1830      1840      1850      1860      1870      1880      1890
1801     CTAGCAGCTGCTTGCTGGCCGCACAACTCACCGGTTCTCAAAGATGTCTCTGTAAGCAAACCAGACAACCTTGTTGCAGCATCAAGCAGT
601       L  A  A  A  C  W  P  H  N  S  P  V  L  K  D  V  S  V  S  K  P  D  N  L  V  A  A  S  S  S

               1900      1910      1920      1930      1940      1950      1960      1970      1980
```

```
1891     GTTCCTCCCAAGCTTGTGATCCTCCACATGAACATGGCCCTCTATGTATATGATGATATAATGAGGATGACTGAAAATTTGAGTGAAAAA
631       V  P  P  K  L  V  I  L  H  M  N  M  A  L  Y  V  Y  D  D  I  M  R  M  T  E  N  L  S  E  K

                  1990      2000      2010      2020      2030      2040      2050      2060      2070
1981     TACATTATTGGTTATGGAGCATCAAGTACTGTCTACAGATGTGATCTGAAGAACTGCAAGTCAGTCGCGATAAAAAAGCTATATACTCAC
661       Y  I  I  G  Y  G  A  S  S  T  V  Y  R  C  D  L  K  N  C  K  S  V  A  I  K  K  L  Y  T  H

                  2080      2090      2100      2110      2120      2130      2140      2150      2160
2071     TACCCGCAGAGCTTGAAGGAATTTGAGACCGAACTTGAGACTGTTGGGAGCATCAAACACCGAAATCTCGTCAGCCTTCAGGGGTACTCC
691       Y  P  Q  S  L  K  E  F  E  T  E  L  E  T  V  G  S  I  K  H  R  N  L  V  S  L  Q  G  Y  S

                  2170      2180      2190      2200      2210      2220      2230      2240      2250
2161     CTGTCACCTGCTGGGAATCTCCTCTTCTATGATTACATGGAAAATGGCAGCCTCTGGGACGTTTTGCATGTAGCTTCATCCAAGAAGGAA
721       L  S  P  A  G  N  L  L  F  Y  D  Y  M  E  N  G  S  L  W  D  V  L  H  V  A  S  S  K  K  E

                  2260      2270      2280      2290      2300      2310      2320      2330      2340
2251     AAACTTGATTGGGAAGCACGCCTCAAGATTGCTCTTGGTGCTGCTCAAGGCCTGGCTTATCTTCACCACGAATGCAGTCCGCGAATAATT
751       K  L  D  W  E  A  R  L  K  I  A  L  G  A  A  Q  G  L  A  Y  L  H  H  E  C  S  P  R  I  I

                  2350      2360      2370      2380      2390      2400      2410      2420      2430
2341     CACAGGGATGTAAAGTCAAAGAATATCCTCCTAGACAAAGACTATGAAGCTCATCTTGCTGACTTTGGTATTGCCAAGAGCTTGTGTGTG
781       H  R  D  V  K  S  K  N  I  L  L  D  K  D  Y  E  A  H  L  A  D  F  G  I  A  K  S  L  C  V

                  2440      2450      2460      2470      2480      2490      2500      2510      2520
2431     TCTTTGACACACACATCAACATATGTAATGGGCACCATTGGCTACATTGATCCTGAGTATGCACGCACATCCCGGCTCAATGAGAAATCA
811       S  L  T  H  T  S  T  Y  V  M  G  T  I  G  Y  I  D  P  E  Y  A  R  T  S  R  L  N  E  K  S

                  2530      2540      2550      2560      2570      2580      2590      2600      2610
2521     GATGTATACAGCTATGGCATCGTCTTGCTGGAGTTGCTTACTGGCAAAAAGCCCGTCGACGATGAGTGCAATCTTCATCACTTGATCCTA
841       D  V  Y  S  Y  G  I  V  L  L  E  L  L  T  G  K  K  P  V  D  D  E  C  N  L  H  H  L  I  L

                  2620      2630      2640      2650      2660      2670      2680      2690      2700
2611     TCCAAAGCTGCAGACAACACGGTCATGGAGATGGTTGACCCAGACATCACGGACACGTGCAAAGATCTTGGCGAGGTCAAGAAGGTGTTC
871       S  K  A  A  D  N  T  V  M  E  M  V  D  P  D  I  T  D  T  C  K  D  L  G  E  V  K  K  V  F

                  2710      2720      2730      2740      2750      2760      2770      2780      2790
2701     CAGTTGGCACTCCTTTGCAGCAAGAGGCAACCATCGGATCGACCGACGATGCATGAGGTCGTGCGCGTCCTGGACAGCCTAGTCTGCCCA
901       Q  L  A  L  L  C  S  K  R  Q  P  S  D  R  P  T  M  H  E  V  V  R  V  L  D  S  L  V  C  P

                  2800      2810      2820      2830      2840      2850      2860      2870      2880
2791     GACCCGCCCCCAAAGCAGGCACAGCCACAGGGATCGGAACAGTCAGCCACAGCTCCGAGCTACGTCAGTGAGTATGTCAGCCTACGAGGC
931       D  P  P  P  K  Q  A  Q  P  Q  G  S  E  Q  S  A  T  A  P  S  Y  V  S  E  Y  V  S  L  R  G

                  2890      2900      2910      2920      2930      2940      2950      2960      2970
2881     GGCACCGCGCTCTCCTGTGCCAATTCATCGAGTGCGTCCGATGCCGAGCTCTTCATGAAGTTTGGCGAGGTGATATCACGGAACACAGAA
961       G  T  A  L  S  C  A  N  S  S  S  A  S  D  A  E  L  F  M  K  F  G  E  V  I  S  R  N  T  E

2971     TAA
991       *
```
