# Supplementary figures and images for: Enhanced biomass and thermotolerance of Arabidopsis by SiERECTA isolated from Setaria italica L
Source: PeerJ. 2022 Dec 1;10:e14452. doi: 10.7717/peerj.14452 (PMC9744159; doi:10.7717/peerj.14452)

**Annex 5 Figure S2** The conserved structure domain of SiER family members

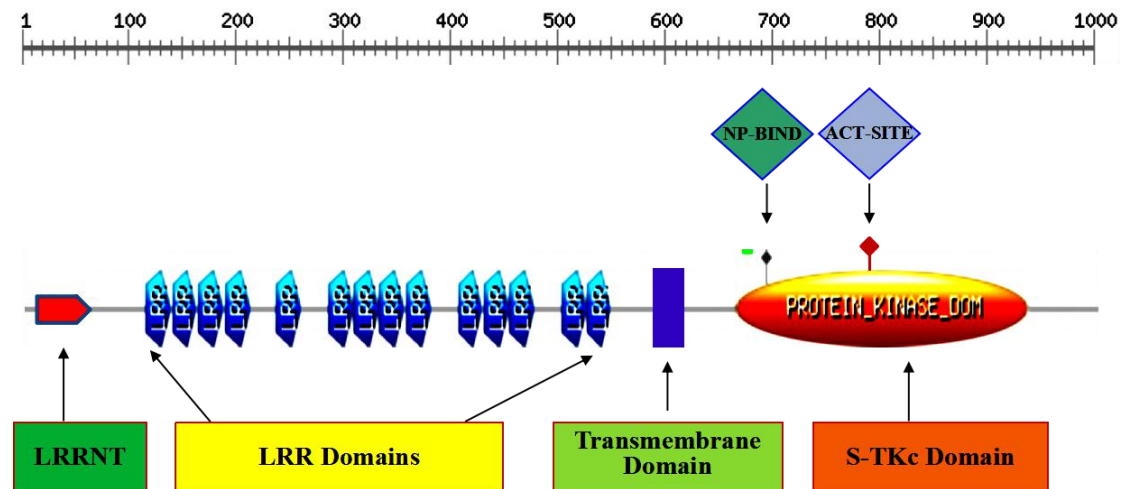

Supplement: Supplemental Information 5 [file peerj-10-14452-s005.pdf]

**Annex 7 Figure S4** Isolation of SiER1\_X4 and SiER4\_X1 genes

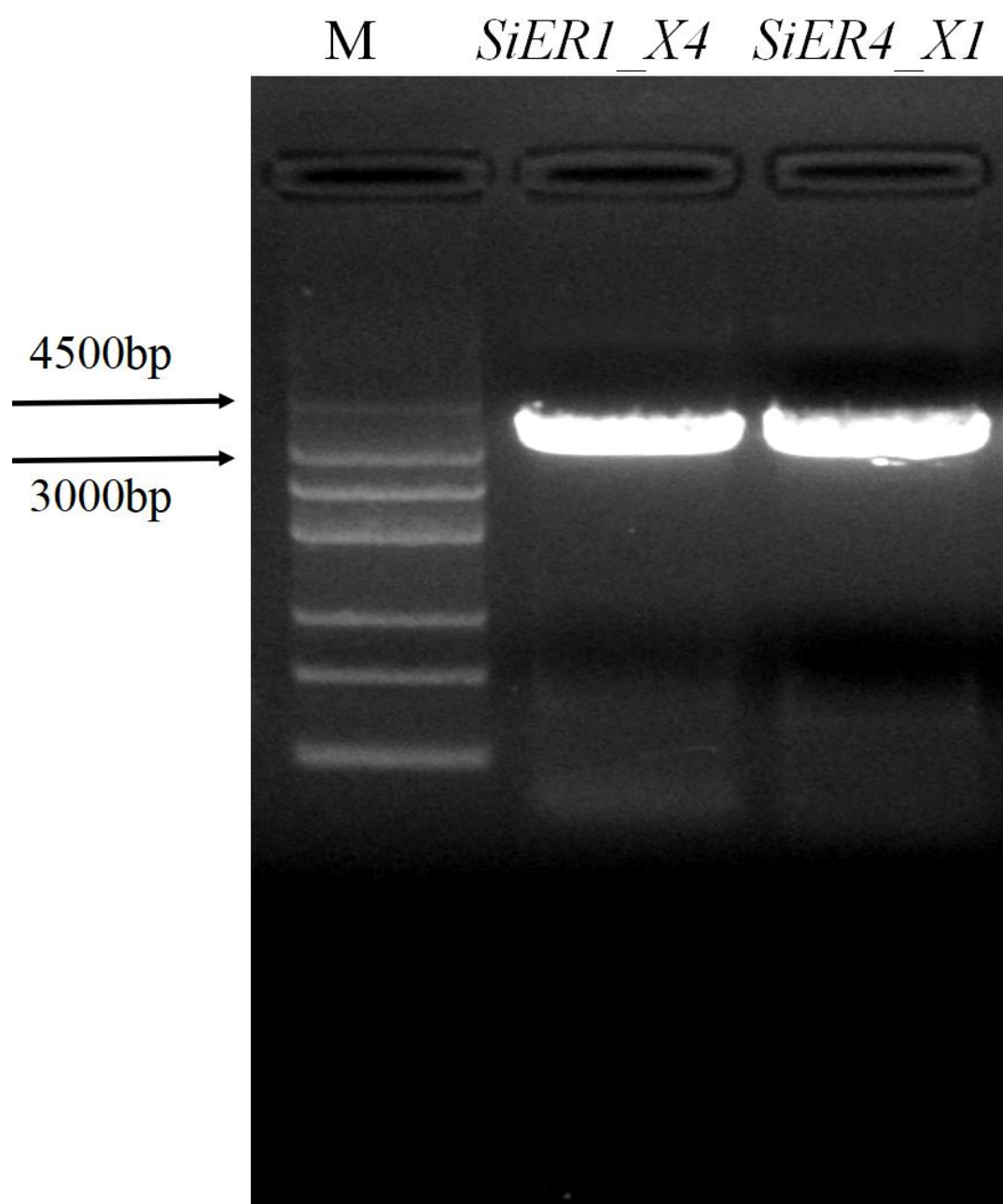

Supplement: Supplemental Information 7 [file peerj-10-14452-s007.pdf]
