# Supplementary material for: Enhanced biomass and thermotolerance of Arabidopsis by SiERECTA isolated from Setaria italica L
Source: PeerJ. 2022 Dec 1;10:e14452. doi: 10.7717/peerj.14452 (PMC9744159; doi:10.7717/peerj.14452)

Annex 6 Figure S3 Alignment of ERECTA family in N-terminal and transmembrane domains

(A) N-terminal signal peptide

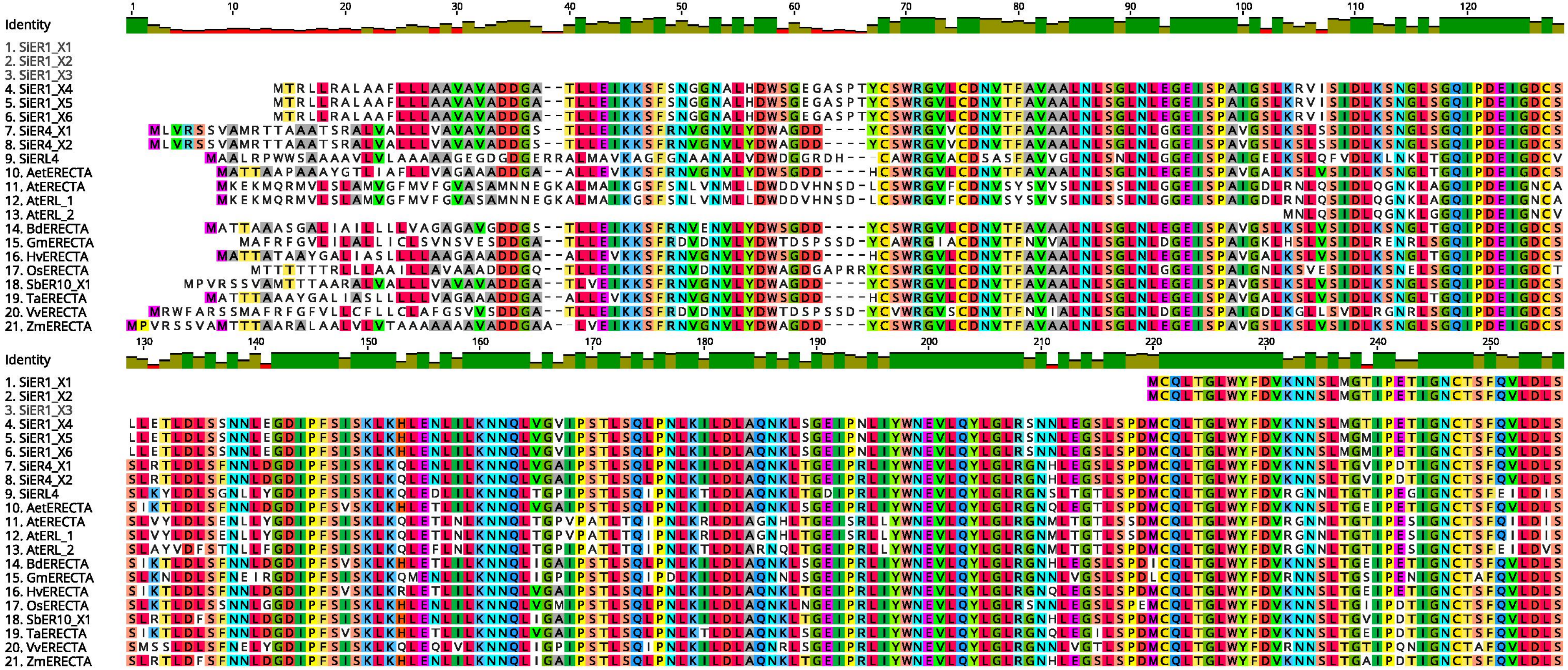

(B) Transmembrane Domain

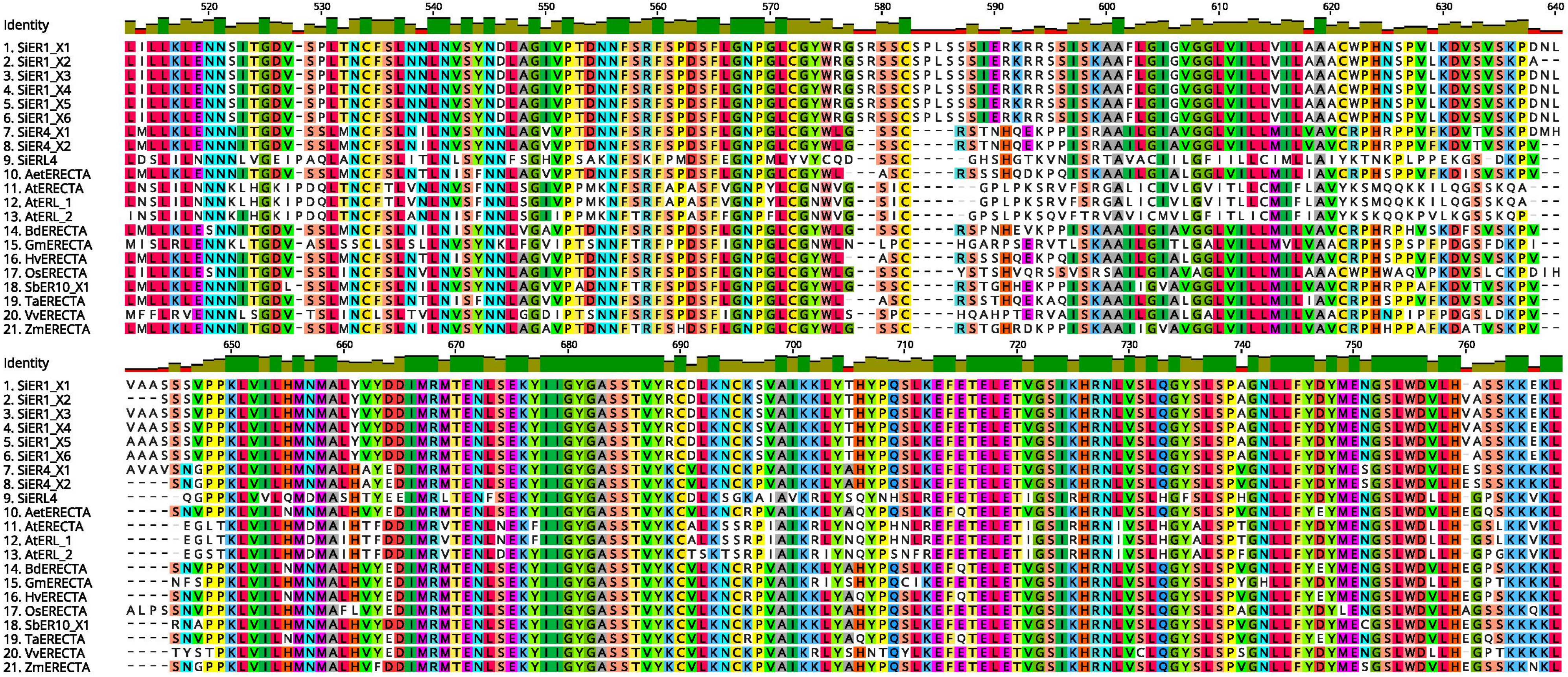

Supplement: Supplemental Information 6 [file peerj-10-14452-s006.pdf]
